# Singlet oxygen luminescence detector based on low-cost InGaAs avalanche photodiode

**DOI:** 10.1016/j.ohx.2021.e00224

**Published:** 2021-08-17

**Authors:** Alexander E. Moskalensky, Tatyana Yu. Karogodina, Alexey Yu. Vorobev, Sergei G. Sokolovski

**Affiliations:** a Novosibirsk State University, Novosibirsk, Russia; b Aston Institute of Photonic Technologies, Aston University, Birmingham, UK

**Keywords:** Singlet oxygen luminescence, Photosensitizers, Avalanche photodiode

## Abstract

Molecular oxygen excited to singlet state (Singlet oxygen, ^1^O_2_) becomes highly reactive and cytotoxic chemical. ^1^O_2_ is commonly generated by photoexcitation of dyes (photosensitizers), including the photodynamic therapy and diagnostics of cancer. However, the formation of singlet oxygen is often unwanted for various light-sensitive compounds, e.g. it causes the photobleaching of fluorescent probes. In either case, during a development of new photosensitive chemicals and drugs there is a need to evaluate the amount of ^1^O_2_ formed during photoexcitation. The direct approach in measuring the amount of singlet oxygen is based on the detection of its luminescence at 1270 nm. However, this luminescence is usually weak, which implies the use of highly sensitive single-photon detectors. Thus the existing instruments are commonly complicated and expensive. Here we suggest an approach and report a device to measure the ^1^O_2_ luminescence using low-cost InGaAs avalanche photodiode and simple electronics. The measurements can be performed in stationary (not time-resolved) mode in organic solvents such as tetrachloromethane (CCl_4_), ethanol and DMSO. In particular, we performed spectral-resolved measurements of the singlet oxygen luminescence in CCl_4_ with the device and demonstrated high complementarity to literature data. The simple setup allows to evaluate the efficiency (or speed) of singlet oxygen generation and hence facilitates the development and characterization of new photosensitizers and other photosensitive chemicals.

## Graphical abstract

**Figure:**
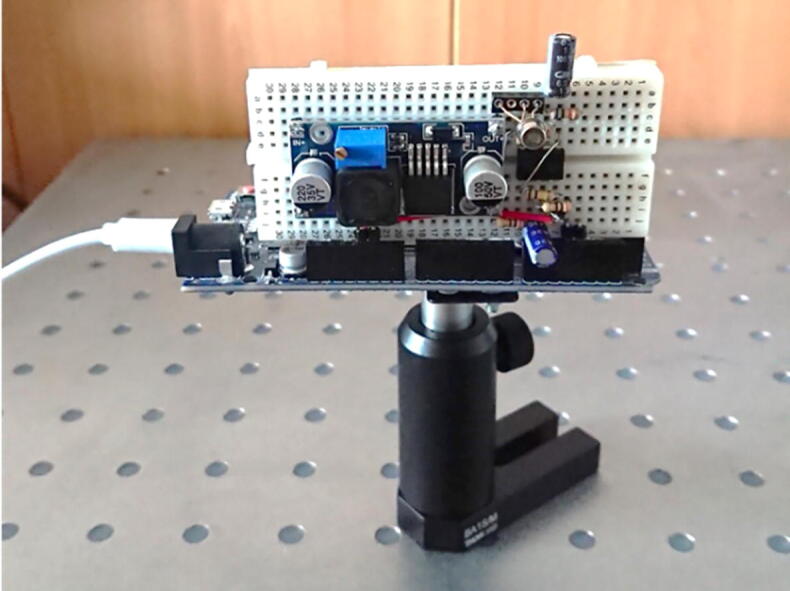

**Table:** 

Hardware name	Simple and inexpensive detector of singlet oxygen luminescence
Subject area	• Biophysics • Chemistry and Biochemistry • Medical (e.g. Pharmaceutical Science)
Hardware type	• Measuring physical properties and in-lab sensors
Open Source License	CC-BY 4.0
Cost of Hardware	$165
Source File Repository	Everything is included in the body of the manuscript

## Hardware in context

In its singlet state the molecular oxygen is extremely reactive and can effectively oxidate compounds crucial in cell biology and chemical reagents. It is known that this excited molecule is the main active agent in many photodynamic reactions in chemical and biological systems. Singlet oxygen is formed during photoexcitation of pigments (porphyrins, chlorophylls etc.) and synthetic dyes (for instance, Rose Bengal) under aerobic conditions [1], [2]. Photodynamic therapy of cancer tumors and inactivation of pathogens is based on photosensitizers, a family of chemicals whose activation by light results in the generation of reactive oxygen species (ROS) including ^1^O_2_ [3]. However, in many other cases singlet oxygen and its ROS derivates have negative, undesirable effect in breaking organic materials, terminating chemical reactions and therapeutic effects of the light-triggered drugs [4] and causing the photobleaching of fluorescent probes. Thus, to optimize the molecular design of the light-responsive chemicals, it is necessary to measure the efficiency of singlet oxygen generation upon photoexcitation.

The most direct way to estimate ^1^O_2_ is to measure its luminescence at 1270 nm. In an aqueous medium, the intensity of this luminescence is very low, and therefore photomultiplier tubes (sometimes with cooling) are used for its detection in the single-photon counting mode. This leads to the fact that instruments for measuring the luminescence of singlet oxygen are complex and expensive [5], [6]. That is why many laboratories use indirect methods for ^1^O_2_ estimation, such as fluorescent sensors, for instance, the Singlet Oxygen Sensor Green (SOSG) [7], [8], [9].

On the other hand, advances in semiconductor technology have led to the emergence of sensitive detectors, which can be used for the detection of ^1^O_2_ luminescence. Mizumoto et al [10] used InGaAs photodiode cooled to 77 K with charge integration system. Boso et al [11] described the negative-feedback avalanche diode operating in single photon mode, capable of the ^1^O_2_ luminescence detection in biological medium. Finally, large-area photodiode can be used for ^1^O_2_ dosimetry [12], [13], including the time-resolved measurements [14]. Avalanche photodiodes, despite relatively small active area, have internal amplification mechanism, which provides superior signal-to-noise ratio and even (in special circumstances) enables single-photon detection. These devises also have great potential for manufacturing arrays and matrices, and some of them are available on the market. In recent years, ultra-cheap infrared avalanche photodiodes have appeared. In this paper, we show that such a photodiode can be used for measuring the luminescence of singlet oxygen and provide the detailed instructions how to do so.

## Hardware description

The sensitivity of commercially available (very expensive) devices measuring the ^1^O_2_ luminescence is excessively high for many practical tasks. In particular, if one aims to characterize the ability of newly synthesized substances to produce the singlet oxygen, there is no need to carry out time-resolved measurements, and it is also possible to use solvents in which the luminescence intensity is significantly higher than in water, in particular, tetrachloromethane / carbon tetrachloride (CCl_4_) [15], [16], ethanol, DMSO and D_2_O. Targeting above problems, the purpose of our study is to make reliable and simple photonic setup for the characterization of novel compounds in their ability to generate the singlet oxygen. Therefore, we hope that the device developed and described here would help researchers to develop novel photosensitizers and light-responsive drugs.

The key features of the setup are as follows:

• The detector have pW sensitivity and is able to detect ^1^O_2_ luminescence;
• The setup is very cheap, so each laboratory that aims to measure in ^1^O_2_ can have it;
• No expensive consumables are required for the measurements.

## Design files

**Fig. 2:**
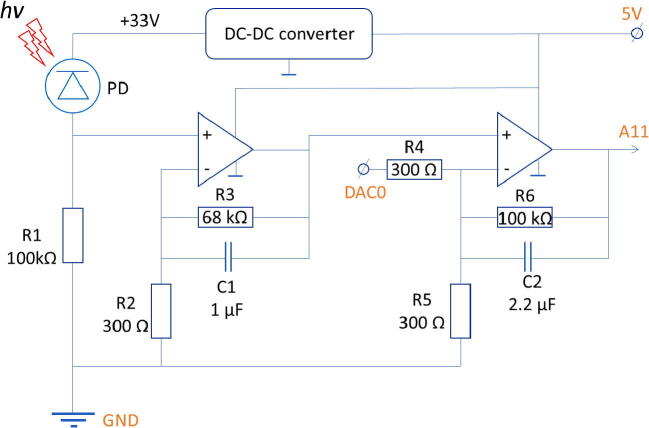
The schematic diagram. PD – photodetector. The points which should be connected to Arduino DUE pins are marked by the corresponding orange text. (For interpretation of the references to colour in this figure legend, the reader is referred to the web version of this article.)

The wiring diagram (Fig. 2) provides the necessary information on how to connect all the components to make a ready-to-use detector. As the diagram is rather simple, the reader can use solderless board, solderable breadboard or any other board of choice. The firmware for Arduino DUE provides the reading of inputs, compensation for DC component and data transfer to the PC. The Python reading script receives data from the detector, makes a graph and allows one to set hardware parameters such as background level and the illumination LED state (on/off).

## Bill of Materials

**Fig. 1:**
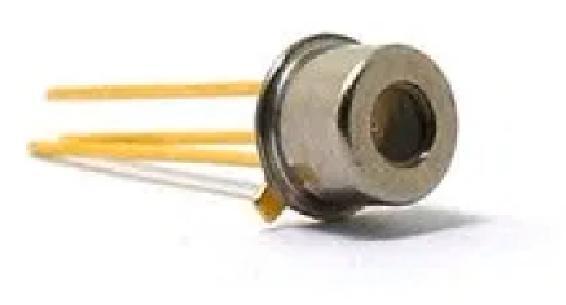
The LSIAPD-200 avalanche photodiode.

Our setup utilizes the low-cost avalanche photodiode LSIAPD-S200 Fig. 1, with following key parameters:

• Material: InGaAs
• Wavelength range: 800–1700 nm
• Photosensitive area diameter: 200 um
• Breakdown voltage V_br_: ~30 V
• Avalanche multiplication gain M: 10 at V_br_ − 4, 30 at V_br_ − 1 (1550 nm)
• Sensitivity (not amplified, M = 1): 0.85 mA/mW @ 1550 nm
• Dark current: 10–30nA at M = 10.

To our knowledge, the only place where this photodiode is available is Aliexpress (see two different links in the table).

Other main building blocks are the Arduino DUE microcontroller board, which reads the data and transfer it to PC, the DC-DC converter which sets the reverse bias voltage for the photodiode, and the operational amplifier. The amplifier is made around the LM-358 integrated circuit using several simple components (resistors and capacitors) which are listed in the table. For these components, marked as “elementary parts” in the table, probably the best source is the local electronics store. Therefore, the prices in the table are only approximate.

## Build instructions

### Basic structure

**Fig. 3:**
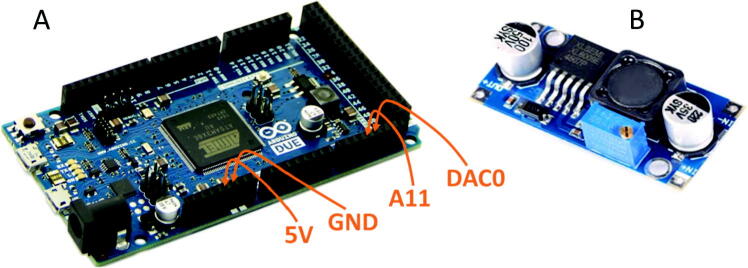
A: Arduino DUE microcontroller board and the inputs/outputs used in the described setup. B: DC-DC converter which provides the reverse bias voltage for the photodiode.

The schematic diagram is shown in the Fig. 2. We have chosen the Arduino DUE board (#2 in Table 2) because of two reasons. First, it has 12-bit resolution of ADC and so provides better precision. Second, it has DAC which is used to compensate for the background component of detector photocurrent. This ability is critical because it eliminates the need to control environmental parameters such as temperature so that the dark current would be fixed. Instead, the influence of external parameters are compensated using active circuit: for each measurement the Arduino board compares the signal with upper and lower bounds (set as 4000 and 500, respectively) and increases/decreases the DAC output if necessary (see the firmware file, #2 in Table 1). This solution is much simpler than the temperature control. The board is connected to the main circuit by four pins (Fig. 3A) and to the PC via USB cable.

**Table 1:** Design Files Summary.

Design file name	File type	Open source license	Location of the file
Schematic diagram (Fig. 2)	image	CC BY 4.0	available with the article
Arduino Firmware	.ino	CC BY 4.0	available with the article
Python script to read data from PC	.py	CC BY 4.0	available with the article

**Table 2:** Bill of materials.

Designator	Component	Number	Cost per unit -currency	Total cost - currency	Source of materials	Material type
Detector	Avalanche photodiode LSIAPD-S200	1	$108-$150	$110.92	https://aliexpress.ru/item/4000515543491.html https://aliexpress.ru/item/32842314109.html	semi-conductor
Electronics – building block	Arduino Due	1	$40.30	$40.3	https://store.arduino.cc/usa/due	other
Electronics – building block	XL6009 DC-DC boost module (converts 5 V DC to ~ 30–40 V DC)	1	$4.5	$4.5	https://www.chipdip.ru/product/xl6009-dc-dc-module	other
Electronics – elementary part	LM358 dual operational amplifier integrated circuit	1	$0.2	$0.2		semi-conductor
Electronics – elementary part	Resistor, 100 k Ohm 0.25 W	2	$0.04	$0.08		other
Electronics – elementary part	Resistor, 68 k Ohm 0.25 W	1	$0.04	$0.04		other
Electronics – elementary part	Resistor, 300 Ohm 0.25 W	3	$0.04	$0.12		other
Electronics – elementary part	Capacitor, 1 uF	1	$0.1	$0.1		other
Electronics – elementary part	Capacitor, 2.2 uF	1	$0.06	$0.06		other
Electronics – housing (optional)	Solderless breadboard	1	$7.5	$7.5		other
Electronics – housing (optional)	PLS-40R right angle pin	1	$0.2	$0.2		other
			TOTAL:	$164.72

The DC-DC converter (#3 in Table 2) should output the regulated voltage of around 30–40 V with the input voltage of 5 V. It is the only important feature, the reader can use any converter which can do the job. The current would be very small, so the maximal output power is not significant. Much of the converters would look like the one we used (Fig. 3B).

### Assembling the amplifier

**Fig. 4:**
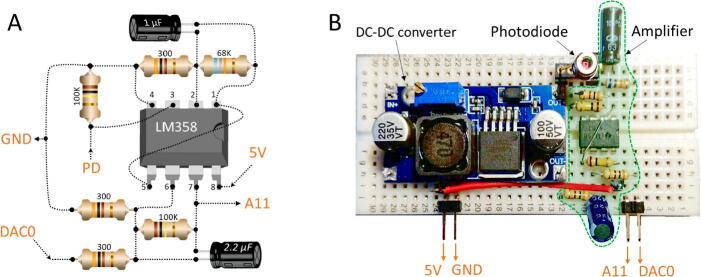
A: The wiring diagram showing how to assemble the amplifier. B: The detector/electronics unit ready to connect to the Arduino DUE board.

The amplifier is probably the most complicated part of our setup. It is based on the LM358 integrated circuit. The wiring diagram is shown in Fig. 4A. The reader may prefer any connection approach (for instance, soldering board). Fig. 4B shows the whole electronic unit assembled on a solderless breadboard, and below are the assembling instructions.

Place the LM358 on the breadboard as shown in Fig. 4B.
Connect the 68 K Ohm resistor to #1 and #2 pins of LM358 (lines #7 and #8 in the upper half).
Connect the 1 µF capacitor to same lines. The capacitor is needed to filter out AC noises as we are interested only in the stationary signal.
Connect the 300 Ohm resistor to #2 and #4 (ground) pins of LM358 (lines #8 and #10 in the upper half). Now you have assembled the feedback loop for the first amplifying cascade.
Pin #3 will accept the signal from the photodiode. Leave the hole on top of line #9 for this purpose. Connect this line to ground (pin #4) via 100 K Ohm resistor.
Connect the #1 and #5 pins of LM358 by a jumper wire (line #7 in the upper half to line #10 in the lower half). Now you have connected the output of the first amplifying cascade to the input of the second one.
However, the output contains significant background component which should not be amplified (otherwise the result would be above threshold of the amplifier). To subtract the background component, connect the #6 pin of LM358 to the DAC0 pin of Arduino DUE (lines #5 and #9 in the lower half) via 300 Ohm resistor.
Connect the 100 K Ohm resistor to #6 and #7 pins of LM358 (lines #8 and #9 in the lower half).
Connect the 2.2 µF capacitor to same lines.
Connect the #6 pin of LM358 (line #9, lower half) to line #12 (lower half) via 300 Ohm resistor.
Connect the #12 line (lower half) to the GND (#22 line, lower half). Also connect this line to #11 and #10 lines on the upper half to provide ground for the LM358 and the photodiode.
Finally, connect the +5 V Arduino pin and #8 pin of LM358 (line #23 and #7, lower half).

### Connecting the photodiode

The avalanche photodiode needs the reverse bias voltage to function. The reverse bias voltage should be in order of V_br_ − 4 to V_br_ − 1, where V_br_ is the breakdown voltage (typically 30–40 V, written on the package of photodiode). Connect the + 5 V Arduino pin with line #28 (upper half) and the GND pin to its lower half. Then place the DC-DC converter to the breadboard. You may need to solder pins to its inputs and outputs. Place the IN + pin to line #28 (upper half), OUT + pin should be connected to line #12 (upper half), OUT − pin would be also on line #12 (lower half). Caution: avoid short-circuit because line #11 is very close and is connected to ground. You need to adjust the output voltage of DC-DC converter to the appropriate level (e.g., 30 V). For this purpose, connect the +5 V and GND pins to the 5 V power source and measure the output voltage. Adjust the screw on the blue resistor to get the desired output. Disconnect the power source.

Now it is ready to connect the photodiode. It has three pins: case, anode and cathode. Carefully connect cathode to the OUT + pin of DC-DC converter (line #12, upper half), case to ground (line #11, upper half) and anode to the #3 pin of LM358 (line #9, upper half). Remember that the photodiode is sensitive to static charges.

### Setting up the Arduino board

**Fig. 5:**
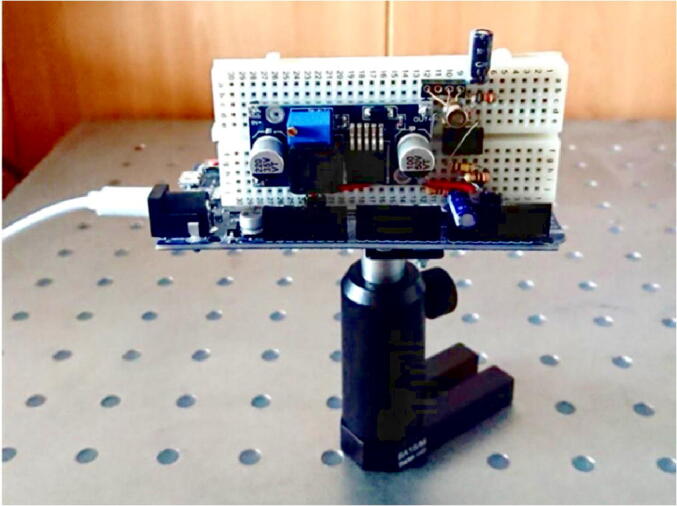
Fully assembled setup.

To connect the detector/electronics unit to the Arduino board, insert right-angle connectors to lines #5, 6, 22 and 23 in the lower half. Before connection, load the firmware (Table 1) from your PC (for the instructions please refer to the specific Arduino resources into the Internet). You may also want to fasten the board to some holder, for instance, we used Thorlabs post and post holder for this purpose. Finally, connect all that you have assembled in previous steps to the Arduino board. Fig. 5 shows how the fully assembled setup looks like in our laboratory.

## Operation instructions

The setup should be kept in dark place during operation (otherwise the weak luminescence signal cannot be measured). To avoid the influence of visible light, it is better to use infrared filter (for instance, 1–1.5 µm bandpass) in all experiments. Additionally the detector can be preceded by monochromator (if one needs to measure the spectrum) or the optical filter specifically for the ^1^O_2_ luminescence [17].

As the setup is connected to PC via USB cable, it continuously measures the signal from photodiode. It is visible in the system as COM-port and is ready to perform the following actions on demand:

• If the PC sends “r”: transfer current detector output to the PC.
• If the PC sends “d”: decrease the background level.
• If the PC sends “u”: increase the background level.
• If the PC sends “l”: enable pin 31 (logical 1, HIGH).
• If the PC sends “o”: disable pin 31 (logical 0, LOW).

The last two commands are needed to switch the excitation light source on/off. For instance, you can control laser or LED-based devise with the corresponding input or you can assemble you own circuit which uses high-current electronic switch opened/closed by the Arduino output (pin #31). Connection of light source directly to Arduino output is not recommended because it would consume current and change the voltage on DAC0 pin, shifting the background level.

**Fig. 6:**
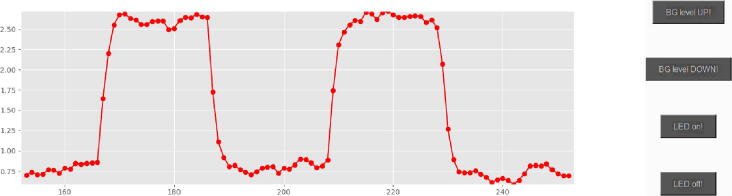
Screenshot of the Python program for data reading and control of the setup.

The Python script which manages the setup is provided with this paper (#3 in Table 1). It reads the detector value every 0.2 s, makes a real-time plot (last 100 points) and stores all data to a specified file. The user will need only to change the number of COM port because it differs between systems and set the filename before measurements. The screenshot of the program is shown in Fig. 6. As it can be seen, the program has very basic interface, but the user can easily add what is needed for the particular experiment. The capability of Arduino board to switch the excitation light source on/off allows one to make an automated measurement procedure so that the difference between signals before/during illumination would be calculated by the script. In our case, we also performed the monochromator control by the same script to achieve an automated measurement of the luminescence spectrum.

## Validation and characterization

### Spectral-resolved measurements

**Fig. 7:**
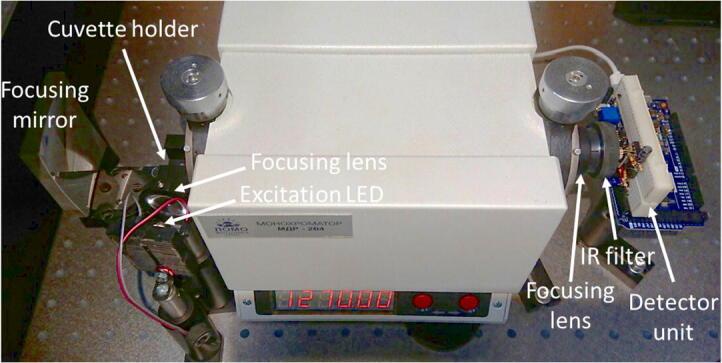
Experimental setup for spectral-resolved measurement of singlet oxygen fluorescence.

To test our setup in real-life experiments, we first assembled the optical setup which includes monochromator, focusing optics, excitation LED (525 nm, 3 W) and cuvette holder (Fig. 7). The excitation LED was connected to the electronic switch controlled by the Arduino board so that we could switch it on/off from the PC.

The MDR-204 monochromator used in this work have an f-number *f*/6. Both slits were opened to 5 mm. We placed the cuvette directly next to the entrance slit, and the luminescence was additionally collected by concave mirror with 10-cm focal length. Alternatively, in some experiments we placed the condenser lens (Thorlabs ACL2520, 25 mm diameter, 20 mm focal length) between the cuvette and the entrance slit. Another condenser lens was placed between the exit slit and the detector, which was either the same Thorlabs ACL2520 or Avantes COL-UV/VIS (6 mm diameter, 8.7 mm focal length). The positions of the detector and all other elements were carefully adjusted to provide the maximal signal.

First, the monochromator was set to 1270 nm. There was no reaction of the detector to LED light in the absence of the cuvette or in presence of the cuvette with solvent. However, we observed significant signal for cuvette with the air-equilibrated solution of Rose Bengal in CCl_4_ (~80 µM).

**Fig. 8:**
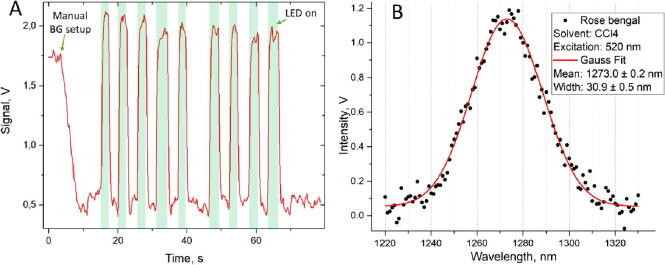
A: the representative recording of the singlet oxygen luminescence during photoexcitation with green LED (525 nm) of the saturated solution of Rose Bengal. B: Spectrum of the measured luminescence signal and Gaussian fit. (For interpretation of the references to colour in this figure legend, the reader is referred to the web version of this article.)

Fig. 8A shows the observed data. The first rapid decrease is due to the manual background level setup using the corresponding button in the PC software (Fig. 6). If the background is low, the measurements can be performed without saturation. Upon turning on the excitation LED, the gradual rise of the signal can be seen. The rise time depends on the capacitors used in the amplifier circuit.

In order to prove that the observed signal is indeed the luminescence of singlet oxygen generated by the Rose Bengal photoactivation with green LED, we performed spectral-resolved measurement. Fig. 8B shows the obtained intensity *versus* wavelength and the Gaussian fit. This result perfectly agrees with the literature data [18].

To estimate the sensitivity of our detector, we tried to measure the same signal as in Fig. 8A with the Thorlabs PDA30B-EC detector (Ge Switchable Gain Detector, up to 70 dB amplification). The resulting signal was ~ 1 mV, which according to the parameters of the detector allows us to estimate the luminescence intensity as 2 × 10^-10^ W and the sensitivity of our detector as 1–10 pW. This is quite a poor sensitivity compared to many commercially available detectors (see e.g. fW sensitivity detector Thorlabs PDF10C used in [13] to measure ^1^O_2_ luminescence in water). But this level of sensitivity already gives the opportunity to make some conclusions from experiments, as we show in the next section.

### Measurements in ethanol and DMSO

**Fig. 9:**
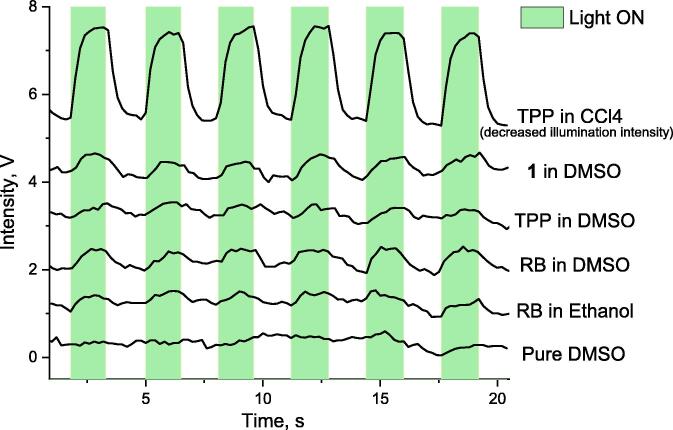
The signals measured with the setup using optical filter instead of monochromator. The plots are vertically shifted for convenience. Top line: tetraphenylporphyrin (TPP) solution in CCl_4_ (the illumination intensity was set to minimal, otherwise the signal was saturated). Other plots are for the solutions of TPP, RB and **1** in other solvents.

As the signal was high enough, we tried to measure the luminescence in solvents other than CCl_4_ because it is more related to the biological system with high water content. First, we modified the measurement scheme, replacing the monochromator by optical filter (Edmundoptics #87–852, 1275 nm central wavelength, 50 nm half-maximum width [17]). Thus, instead of spectral-resolved measurements, we enabled the integration of luminescence signal in a window which corresponds to the peak in Fig. 8B, increasing the signal/noise ration. Moreover, the use of filter allowed us to increase the collection angle, that drastically increased the signal. Similar approach was used in [13]. As a result, we observed the luminescence of ^1^O_2_ during the photoexcitation of Rose Bengal millimolar solution in ethanol and dimethyl sulfoxide (DMSO, a solvent which is frequently used in biological studies to dissolve organic compounds which can then be dissolved in water). The results are shown in the Fig. 9. We also studied tetraphenylporphyrin (TPP) solution in DMSO using the appropriate excitation wavelength (430 nm). All the samples were air-equilibrated. While the signals are quite low, those can be accumulated over many runs to obtain reasonable signal-to-noise ratio. The bottom plot in the Fig. 9 shows the absence of signal in pure DMSO.

**Scheme 1:**
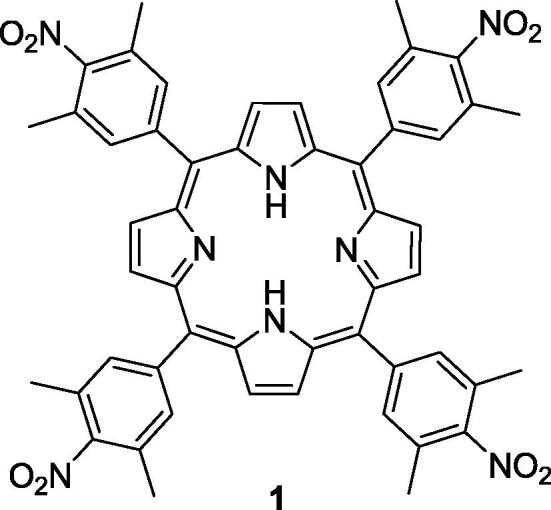
The “unknown” compound **1** used in experiment.

These experiments clearly show that the described setup can provide information on the ability of ^1^O_2_ generation by the compound under study. To demonstrate this, we measured the luminescence during the photoexcitation of porphyrin **1** (Scheme 1, [19]), a novel compound with no known data concerning the ^1^O_2_ generation. The absorbance at the photoexcitation wavelength was matched to that for the TPP solution, and the luminescence signal was higher for the compound **1**, indicating that the quantum yield of ^1^O_2_ generation is higher.

Concerning the further improvement of signal-to-noise ratio, larger-area photodiode can be used (LSIAPD-S500, 0.5 mm active area diameter). There is also available version of LSIAPD-S200 with built-in transimpedance amplifier (LSIAPDT-S200-155M-L0A), which potentially provides better signal-to-noise ratio. Another limitation of the current setup is limited dynamic range. In our experience, we had to decrease the excitation LED current to make the signals not saturated for some chemicals. It complicates the intercomparison between samples. This problem can be solved using hardware control of amplification gain and/or the ADC scale. However, in this paper we aimed at the demonstration of the reported approach and tried to make everything as simple as possible, leaving further improvements for the future. As the system is described in detail, it can be easily adjusted for the particular application, including the dosimetry of ^1^O_2_ during photodynamic therapy. Although, for that last application, additional work is needed to improve the sensitivity of the detector to apply it in real systems.

## CRediT authorship contribution statement

**Alexander E. Moskalensky:** Conceptualization, Methodology, Investigation, Writing - original draft. **Tatyana Yu. Karogodina:** Investigation , Writing - review & editing. **Alexey Yu. Vorobev:** Resources. **Sergei G. Sokolovski:** Writing - review & editing.

## Declaration of Competing Interest

The authors declare that they have no known competing financial interests or personal relationships that could have appeared to influence the work reported in this paper.
